# A retrospective study of extrapulmonary tuberculosis in the Khuzestan province of southwest Iran between 2002 and 2023

**DOI:** 10.1186/s12879-024-10386-0

**Published:** 2024-12-25

**Authors:** Mohammad Hashemzadeh, Aram Asareh Zadegan Dezfuli, Nazanin Ahmad Khosravi, Fatemeh Jahangiri Mehr

**Affiliations:** 1 Student Research Committee, Ahvaz Jundishapur University of Medical Sciences, Ahvaz, Iran; 2 Infectious and Tropical Diseases Research Center, Health Research Institute, Ahvaz Jundishapur University of Medical Sciences, Ahvaz, Iran; 3 Department of Microbiology, Faculty of Medicine, Ahvaz Jundishapur University of Medical Sciences, Ahvaz, Iran; 4 Department of Epidemiology and Biostatistics, School of Public Health, Isfahan University of Medical Sciences, Isfahan, Iran

**Keywords:** Extrapulmonary tuberculosis, Epidemiology, *Mycobacterium tuberculosis*, Data analysis

## Abstract

**Background:**

Worldwide, tuberculosis (TB) is among the most common causes of death. To our knowledge, there has been no study showing the prevalence of EPTB in Khuzestan province. Therefore, the objective of this research was to investigate the prevalence of EPTB in patients with or without pulmonary TB in different cities of Khuzestan province from 2002 to 2023. Additionally, the correlation between patient’s gender, and age groups with the disease was also investigated.

**Methods:**

In this retrospective study, the existing records in Tuberculosis Regional Reference Laboratory of Khuzestan province related to patients were used. The research was carried out by investigating the archive information in 19 years (from 1st January 2002 to December 30, 2023). All confirmed cases of EPTB and simultaneous EPTB and PTB, based on laboratory results and medical examination were included in the study. Patients with incomplete information and military TB were excluded from the study. Information collected from patients includes age, gender, involved organ, place of residence, and year of disease.

**Results:**

A total of 12,900 EPTB-related medical records were extracted from Tuberculosis Regional Reference Laboratories in southwest Iran, Ahvaz. After excluding records, 12,836 clinically diagnosed or laboratory-confirmed tuberculosis patients were included in this study, including 5991 patients with simultaneous PTB and EPTB, and 6845 patients with EPTB only. The mean age of male EPTB patients was 37.5 years (SD ± 14.6), while the mean age of male patients with simultaneous PTB and EPTB was 45.8 years (SD ± 15.3). The mean age of female patients with EPTB only, and with simultaneous PTB and EPTB was 31.2 years (SD ± 12.6), and 31.5 years respectively.

**Discussion:**

tuberculosis is a systemic disease with different clinical manifestations. This study described different epidemiologic patterns of concurrent EPTB. The proportion of different types of EPTB was simultaneously determined for a group of hospitalized patients and shown to be different with gender and age. This study will likely increase clinicians’ awareness of the disease and help them better address diagnostic challenges and improve treatment outcomes for patients with EPTB.

**Supplementary Information:**

The online version contains supplementary material available at 10.1186/s12879-024-10386-0.

## Background

Worldwide, tuberculosis (TB) is among the most common causes of death [1]. According to the recent World Health Organization (WHO) report on TB [1], there are currently 11.9 million cases in the world, including 9 million new cases with three million deaths annually [2]. Apart from pulmonary TB, the number of cases of extrapulmonary TB (EPTB) annual deaths has increased worldwide and represents about 12% of all deaths, especially in developing countries [3]. The body’s organ systems are affected by EPTB in a variety of ways [4]. Some forms of EPTB, including meningitis TB and pericarditis TB, have high mortality rates, while other forms of EPTB can cause disability in patients [5]. The disease TB is still considered a serious threat to human life, despite the advent of new diagnostic and treatment methods [6]. In light of the rising incidence of AIDS, the problem of TB is gaining more and more attention [7]. There is also an increase in EPTB which affects 21% of patients with AIDS [8, 9]. A study conducted by the Pasteur Institute of Iran in 2013, revealed that 22.5% of TB cases occurred in the EP organs, including lymph nodes (39%), bone and joint (18%), meninges (16%), urinary system (12%) and other organs (16%) [10, 11]. According to another study, endometrium accounted for 5% and other organs accounted for 15% of EP cases [12]. The most common age is 25 years old (38.9%) under 14 years 16.7% and over 65 [13]. Over 5 years, Bakhtaran examined cases of EPTB, finding that 27% of cases were EP, with tuberculous lymphadenitis again being the most common (42%) [14]. Even though TB is not prevalent throughout the entire country of Iran, it is more prevalent in marginal areas of the country [15]. Khuzestan province is in the southwest of Iran, and due to the endemicity of TB in Iran, it seems necessary to investigate EPTB. To our knowledge, there has been no study showing the prevalence of EPTB in Khuzestan province. Therefore, the objective of this research was to investigate the prevalence of EPTB in patients with or without pulmonary TB in different cities of Khuzestan province from 2002 to 2023. Additionally, the correlation between patients’ gender, and age groups with the disease were also investigated.

## Methods

### Study design and population

In this retrospective study, we utilized the existing records in the Tuberculosis Regional Reference Laboratory of Khuzestan province. The research involved investigating archive information spanning 19 years, from 1st January 2002 to 30th December 2023. The study included all confirmed cases of EPTB (extrapulmonary tuberculosis) and simultaneous EPTB and PTB (pulmonary tuberculosis), based on laboratory results and medical examination. Patients with incomplete information and miliary TB were excluded from the study. Information collected from patients included their age, gender, the affected organ, place of residence, and the year of disease.

## Data analysis and statistics

Collected data for age and gender groups in EPTB patients and EPTB and PTB patients. Logistic regression analysis was performed to compare associations of concurrent EPTB among gender and age groups.Odds ratios (ORs) with 95% confidence intervals (CIs) for age group (< 15 years, 15–24 years, 25–34 years, 35–44 years, 45–54 years,55–64 years and ≥ 65 years) and gender were calculated. Association rules obtained for the various age groups and genders with concurrent EPTB were identified using the Apriori algorithm. Primary analyses were based on tests to detect trends by modelling the ordered age categories as linear terms. *P* < 0.05 was considered to indicate statistical significance. In this study, it was based on the Apriori algorithm. Apriori extracts data for support, and confidence and defines the variables as follows: support = P(A), confidence = P(B|A), and lift = P(A∩B)/[P(A ) *P(B)], where A was the antecedent and B was the consequence. The lift was used to assess the degree of association rules (Tang et al., 2013), whereby a lift > 1 indicates a positive association rule while a lift ≥ 3 indicates a strong association rule. In addition, This study investigated 12,836 patients with different types of EPTB and simultaneous PTB-EPTB. The patient’s records included information regarding the date of referral, age, gender, organ involvement, and ethnicity. To express patients’ characteristics, descriptive statistics including tables and graphs, and to determine the relationship between the findings, statistical tests were used.

## Results

### General information about patients

A total of 12,900 EPTB TB cases were identified from Tuberculosis Regional Reference Laboratories in southwest Iran, Ahvaz. After excluding records, 12,836 clinically diagnosed or laboratory-confirmed tuberculosis patients were included in this study, including 5991 patients with simultaneous PTB and EPTB, and 6845 patients with EPTB only. (Table 1). The mean age of male EPTB patients was 37.5 years (SD ± 14.6), while the mean age of male patients with simultaneous PTB and EPTB was 45.8 years (SD ± 15.3). The mean age of female patients with EPTB only, and with simultaneous PTB and EPTB was 31.2 years (SD ± 12.6), and 31.5 years (SD ± 11.10) respectively.

### Classification of patients based on place of residence

**Fig. 1:**
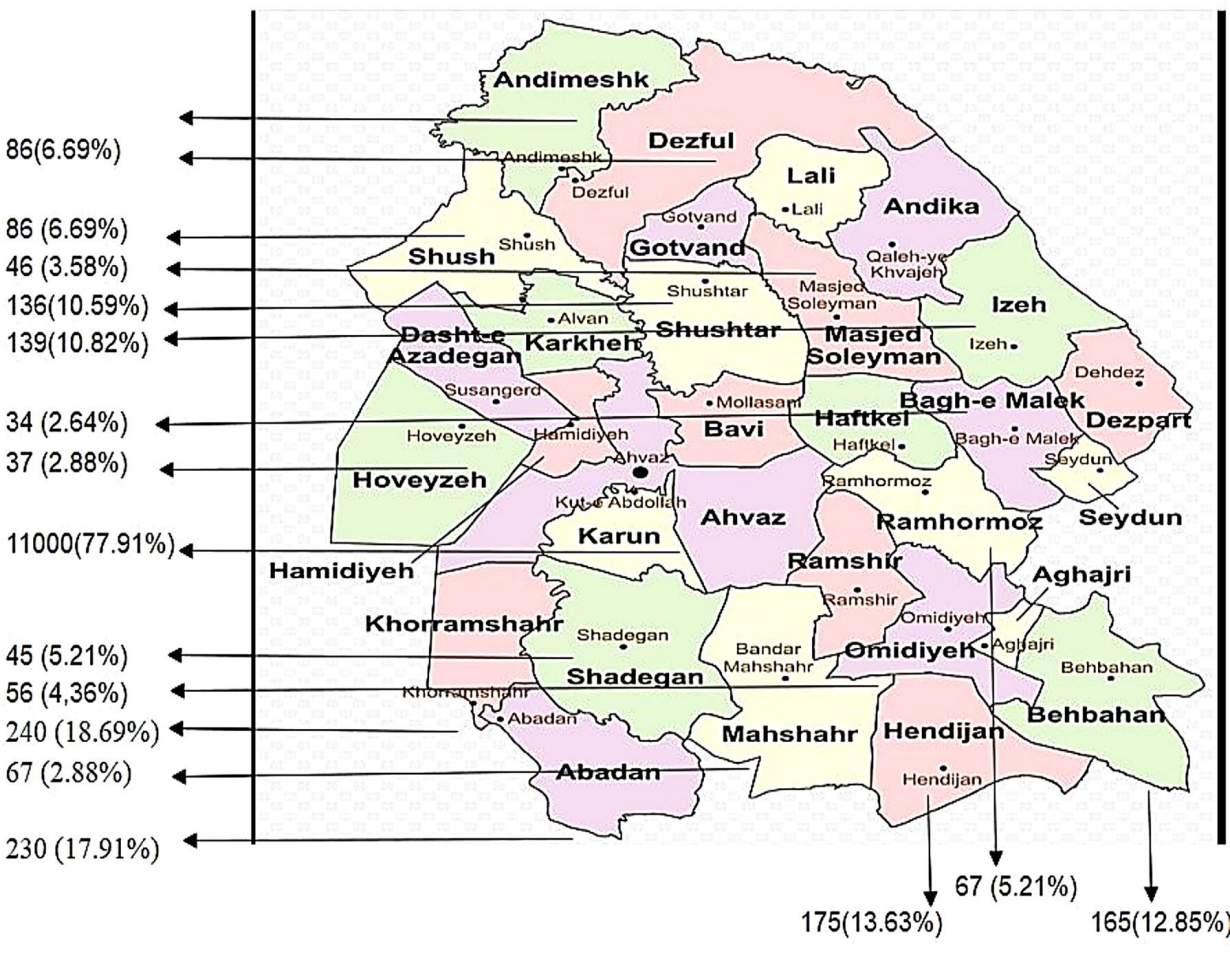
Distribution of pulmonary and extrapulmonary tuberculosis in Khuzestan province

In this study information of patients based on clinical symptoms and laboratory findings including bacteriology, and histology were extracted and collected from different cities of Khuzestan province as follows in Fig. 1:

### Basic characteristics of patients with PTB and PTB-EPTB concurrent

According to the demographic information included in Table 1, out of all 12,836 patients, 6849 (95% CI: 16.2–21. 53.39%) patients were Females, and 5977 (95% CI:53.6–72. 46.67%) patients were Males. The distribution of the age group of patients is shown in Table 1.

**Table 1:** The distribution of the age group of patients

Variables	Total (*n* = 12836)	Proportion (%)
**Gender**		
Female	6859	53.39%
Male	5977	46.67%
**Age group (years)**	**Age group (years)**	**Age group (years)%**
< 15	2454	19.07%
15–25	1125	8.76%
25–35	4087	31.84%
35–45	3000	23.37%
45–55	970	7.55%
55–65	550	4.28%
≥ 65	650	5.06%

**Fig. 2:**
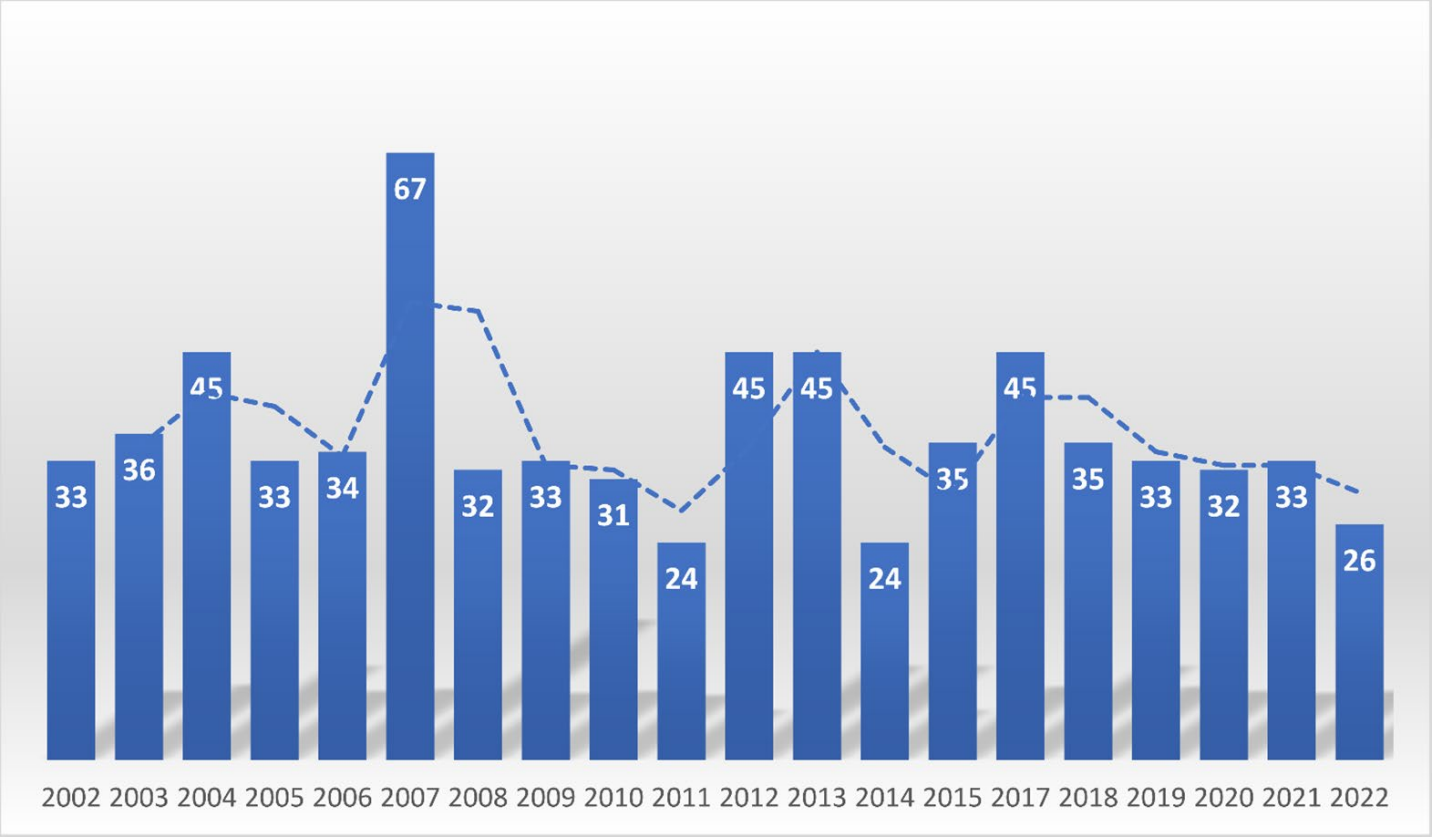
Distribution of extrapulmonary tuberculosis between 2002–2022 in Ahvaz, Iran in moving average chart

No significant difference in EPTB percentage was observed between male and female patients in the two groups. Patients with both EPTB and PTB were most likely to be aged < 15 (34.69). In terms of the distribution of patients in Ahvaz city, 9300 (95% CI: 12.0–15. 72.45) patients were from the East region, while the proportion of the North region of Ahvaz was 700 (95%CI: 19.5–11. 54.53), the West region was 510 (95% CI: 16.8–21. 39.73) and the 490 (95%CI: 19.5–11. 38.17) patients were in central of town. A total of 12,343 (95% CI: 23.1–32. 96.15) cases were laboratory diagnosed as EPTB, which was higher than patients with only clinically diagnosed as EPTB which was as low as 493 (95% CI: 5.2-8. 11.22; aOR: 10.12, 38.40. *P* < 0.001). In terms of the distribution of patients in Ahvaz city, 523 (95% CI: 12.0–15. 19.27) patients were from the Central region, while the proportion of the North region of Ahvaz was 980 (95%CI: 19.5–11. 36.12), the West region was 700 (95% CI: 16.8–21. 25.80) and the 510 (95%CI: 19.5–11. 18.79) patients were in East of town. In EPTB and PTB concurrent, a total of 9735 (95% CI: 74.1–42. 96.16) of cases were laboratory diagnosed, which was higher than patients with clinical diagnosis as 388 (95% CI: 4.5-7. 8.42; aOR: 7.18, 95% CI: 5.76–11.07, *P* < 0.001). Among the patients with EPTB, a total of 2543 patients were(95% CI: 83.10–61. 93.73%) HIV positive. Among the patients with EPTB and PTB concurrent, a total of 8231 (95% CI: 63.01-12. 8130) were HIV positive. A significant difference in EPTB proportion was observed in EPTB-only cases compared with PTB-EPTB concurrent patients (aOR: 2.12, 95% CI: 1.69 − 0.25, *P* = 0.210) (Table 2). In terms of clinical manifestations, the cough was the most common symptom in EPTB patients (aOR: 3.45, 95% CI: 35.34–0.65, *P* = 0.256) and in EPTB and PTB concurrent cases, night sweating was the most common symptom (aOR: 8.34, 95% CI: 65.34–0.89, *P* = 0.254)(Fig. 2).

### Lesion characteristics of patients EPTB

In the current investigation, the most frequent sites of EP involvement were tuberculous lymphadenitis, 1199(44.19) (Table 2). The swelling of lymph nodes was documented in 32/48(66.66) of these patients. Overall, the most common lesion characteristic of EPTB in male patients was tuberculous lymphadenitis 1550(56.52%) (support 63.23, confidence 80.05, lift 73.23**). Similarly, in EPTB female patients, tuberculous lymphadenitis was the most common site 2900(27.58) (support 89.10, confidence 85.94, lift 85.87**) (Appendix Table S2). The proportion of tuberculous lymphadenitis was 57(39.31)in children younger than 15 years (support 76.11, confidence 66.10, lift 74.11**) (Appendix Table S3). In ages 15–25, tuberculous lymphadenitis was the most frequent site 230(44.23) (support 75.12, confidence 70.32, lift 70.11**) appendix Table S4. At the age of 25–35, bronchial tuberculous had the most conflict 158(52.66) (support 87.45, confidence 95.36, lift 85.53**) Appendix Table S5. The proportion of tuberculosis lymphadenitis was the highest in ages 35–45, 247(32.29) (support 85.16, confidence 85.72, lift 85.12**) appendix Table S6. In age 45–55, tuberculous of mediastinal lymph nodes was the most common site 160(35.32) (support 65.21, confidence 65.45, lift 65.23**) appendix Table S7. At the age of 55–65, tuberculosis lymphadenitis had the most rate in patients, 208(56.21) (support 72.95, confidence 72.85, lift 70.23**)(Appendix Table S8). Finally, the proportion of tuberculous lymphadenitis was the highest in patients older than 65 years 135(44.44)(support 74.43, confidence 70.44, lift 10.23**) (Appendix Table S9).

### Lesion characteristics of patients’ PTB and EPTB concurrent

According to the investigated collected data, in PTB and EPTB concurrent patients’ tuberculous lymphadenitis 4440(43.86). Overall, the most common lesion characteristics of PTB and EPTB concurrent, in male and female patients was tuberculosis lymphadenitis with a rate of 1674 (41.44) (support 90.22, confidence 90.43, lift 44.23**), and 2908(71.99) (support 88.65, confidence 88.91, lift 88.23**) respectively (Appendix Table S10 and S11). The proportion of tuberculous meningitis was 600(17.64)in children younger than 15 years (support 77.23, confidence 76.85, lift 75.45**) (Appendix Table S12). In age 15–25 tuberculous lymphadenitis was the most common site 600(17.64) (support 77.23, confidence 76.85, lift 75.45**) Appendix Table S13. In age 25–35, tuberculous lymphadenitis was the most common site 991(66.91) (support 86.87, confidence 88.86, lift 88.78**) Appendix Table S14. The proportion of tuberculous lymphadenitis was 600(33.72) in ages 35–45, (support 86.56, confidence 86.44, lift 86.15**) Appendix Table S15. At the age of 45–55, tuberculous lymphadenitis had the most conflict 990(59.78) (support 89.23, confidence 89.82, lift 89.65**) (Appendix Table S16). At the age of 55–65, renal tuberculosis sites had the most conflict 879(50.92) (support 89.34, confidence 89.54, lift 89.12**) (Appendix Table S17). Finally, the proportion of renal tuberculosis was 338(50.22)in patients older than 65 years (support 58.23, confidence 58.22, lift 58.33**) (Appendix Table S18).

**Table 2:** An analysis of the characteristics of EPTB patients and PTB patients

Characteristics	EPTB (*N* = 2713) %	EPTB with PTB concurrent (*N* = 10123) %	*P*-value
**Gender**			< 0.001
Female	1060(39.07)	6030(59.56)	
Male	1653(60.92)	4039(39.89)	
**Age**			**0.256**
**<** 15	145(10.96)	1467(14.49)	
15–25	520(37.43)	1334(13.17)	
25–35	300(6.95)	1481(14.63)	
35–45	675(11.76)	1786(17.37)	
45–55	453(12.29)	1656(16.35)	
55–65	370(6.41)	1726(17.05)	
**<** 65	250(14.43)	673(65.78)	
**Diagnosis**			**< 0.001**
Laboratory	1278(47.10)	9735(96.16)	
Clinically	1435(52.89)	388(38.32)	
**HIV status**			**0.210**
Positive	2543(93.73)	8231(8130)	
Negative	170(6.26)	1892(18.93)	
**Khuzestan province**			**< 0.001**
Central	523 (19.27)	2310(22.81)	
East	510 (18.79)	990(9.77)	
West	700 (25.80)	4723(46.65)	
North	980 (36.12)	2100(20.74)	
**Documented symptoms**	2713		**0.232**
Night sweating	735(27.9)	1158(11.43)	
Low-grade fever	2345(86.43)	1145(11.31)	
Loss of appetite	864(31.84)	1237(12.21)	
Weight loss	2187(80.611)	1579(15.59)	
Tiredness	690(27.43)	1657(47.55)	
Chest pain	2135(78.69)	1080(1068)	
Cough	2349(78.68)	9678(95.60)	

## Discussion

Despite the recent downward trend in tuberculosis disease, Iran is still considered an endemic region for tuberculosis. It seems that the WHO has not yet reached its goal of controlling tuberculosis, indicating a lack of significant growth. One-third of people with TB can develop extrapulmonary TB (EPTB). The percentage of Iranian TB cases diagnosed with EPTB increased from 20 to 29% in 2022. However, the epidemiology of EPTB is not well understood. This research was the first to reveal the epidemic condition of EPTB in southwest Iran, Ahvaz. The incidence and percentage of EPTB are different in different countries. For example, pleural TB is the most common type of EPTB in China (8.49%), and lymphatic in the USA and Spain (40.4%), (27%) respectively [16–18]. In our study, lymphadenitis tuberculosis and renal tuberculosis had the highest percentage of extrapulmonary tuberculosis. In previous studies, tuberculosis pleuritis is the most common form of EPTB and is the cause of 30–70% of all cases of pleural effusion [19, 20]. These results were not consistent with our study. The difference in results can be caused by the difference in environmental and genetic risk factors between countries, vaccination, sample size, and time of studies [21]. However other studies showed that tuberculosis lymphadenitis is the most common form of extrapulmonary tuberculosis in developing and developed countries [22–25]. These results are similar to our study. Tuberculous meningitis is very important in the central nervous system (especially in children) and has a high mortality rate. Manyelo et al. reported an incidence rate of tuberculous meningitis of 23 (48.9%) among 47 children [26]. A study in Germany showed that tuberculous meningitis was 3.9% in children under 5 years old, 2.2% in children 5–9 years old, and 1.3% in children 10–14 years old [27]. After systematic searching and quality assessment, the overall pooled estimates of CSF culture-positive TBM was 29.72%. The pooled prevalence of MDR-TB among culture-positive TBM cases was 5.19% [28]. In the present study, 225(15.33) of EPTB and PTB concurrent patients younger than 15 years with tuberculous meningitis with a strong association rule (lift = 45.09*) While in EPTB patients younger than 15 years had 14(9.65)tuberculous meningitis with a weak association rule (lift = 70.12*). The presence of tuberculous meningitis, especially in patients who have pulmonary tuberculosis at the same time, can greatly increase the mortality rate in these patients. It may also involve other central nervous systems as the disease progresses. Rupture of tuberculous lesions of the spinal meninges can cause local inflammatory changes,

exudate formation, and subsequent tuberculous spinal arachnoiditis. Considering that tuberculous meningitis is the deadliest type of tuberculosis, therefore, correct and timely diagnosis of this form of the disease is very effective in preventing the death of patients, especially in young patients [29]. In this study, statistical analysis of support, confidence, and lift was used for all patients. From an epidemiological point of view, single-site EPTB is gender-related, with female cases predominating in the United States, Germany, and England, while in our study, single-site EPTB was more common in men [30–32]. In the present study, tuberculous lymphadenitis in ETPB patients with the highest confidence in men as compared to women (843VS 356). Tuberculosis lymphadenitis is very important because it is a hematogenous disease and with descending spread, it can become other forms of tuberculosis including pleural tuberculosis and renal tuberculosis. The values of tuberculous pleurisy lift were higher in women than men (19.13*VS 21.27*), while the values of renal tuberculous lift were higher in women than men (16.45*VS 3.65*). In ETPB and PTB concurrent patients, tuberculous lymphadenitis is the highest confidence in the male it was compared to men (88.91VS 59.23). The values of tuberculous pleurisy lift were higher in men than women (89.43**VS 25.35*), while the values of renal tuberculous lift were higher in women than men (32.34**VS 20.43**).Compared to young and old ages in the study, in patients with EPTB aged 15–25 years, the highest confidence and lift rates were obtained for tuberculous lymph nodes, which have strong association rules (lift = 70.11**), while this pattern was not observed in elderly patients(< 65 years). Instead, in elderly patients (< 65 years), the highest level of confidence and lift was obtained for lymphadenitis tuberculous, which had strong correlation rules (lift = 70.23**,74.43). The mechanism of this difference may be related to BCG vaccination of infants in Iran, which is done to prevent tuberculosis in childhood. However, studies have shown that this vaccine does not provide long-term immunity against tuberculosis. Such age-related increases in TB disease may be related to changes in age-related immunodeficiency. The present findings consider it important to prevent and control EPTB in patients of different ages and to investigate the possibility of aging in old age. It seems that more studies are needed in the future regarding the development of tuberculosis in old age. The incidence of tuberculosis is closely linked to aging and a weakened immune system. As people get older, they become more susceptible to recurring tuberculosis or contracting the disease from an infected individual. People who are already sick are at a higher risk of developing the disease (33–34). Some key strengths of this study include its large sample size, the geographical diversity of patients, detailed statistical analysis, the average duration of the study, and the comparison of age, sex, and organs involved in single-site patients and patients with concurrent tuberculosis for the first time in an endemic region. This study is focused on tuberculosis.

### Limitation

In this study, many Iranian patients had incomplete information, leading to their exclusion from the study. Additionally, non-Iranian diseases were excluded due to incomplete files.

## Conclusion

In conclusion, tuberculosis is a systemic disease with various clinical manifestations. This study has identified different epidemiologic patterns of extrapulmonary tuberculosis (EPTB). The proportion of different types of EPTB has been determined for patients, and it has been shown to vary with gender and age. This study is expected to enhance clinicians’ awareness of the disease and assist them in addressing diagnostic challenges and improving treatment outcomes for patients with EPTB.

## Electronic supplementary material

Below is the link to the electronic supplementary material.

Supplementary Material 1

## Data Availability

All data generated or analysed during this study are included in this published article [and its supplementary information files].

## Abbreviations

TB: Tuberculosis
WHO: The World Health Organization
EPTB: Extrapulmonary TB
AIDS: Acquired immunodeficiency syndrome
PTB: Pulmonary tuberculosis
