# Supplementary material for: A retrospective study of extrapulmonary tuberculosis in the Khuzestan province of southwest Iran between 2002 and 2023
Source: BMC Infect Dis. 2024 Dec 25;24:1463. doi: 10.1186/s12879-024-10386-0 (PMC11670499; doi:10.1186/s12879-024-10386-0)
Supplement: Supplementary file 1 — Supplementary Material 1 [file 12879_2024_10386_MOESM1_ESM.docx]

Supplementary Tables

**Table S1. The lesion characteristics of EPTB in male patients**

**Table S2. The lesion characteristics of EPTB in female patients**

**Table S3. The association rules of EPTB in <15 years patients with EPTB infections**

**Table S4. The association rules of EPTB in 15-25years patients with EPTB infections**

**Table S5. The association rules of EPTB in 25-35 years patients with EPTB infections**

**Table S6. The association rules of EPTB in 35-45 years patients with EPTB infections**

**Table S7. The association rules of EPTB in 45-55 years patients with EPTB infections**

**Table S8. The association rules of EPTB in 55-65 years patients with EPTB infections**

**Table S9. The association rules of EPTB in < 65 years patients with EPTB infections**

**Table S10. The lesion characteristics of EPTB and PTB concurrent in male patients**

**Table S11. The lesion characteristics of EPTB and PTB concurrent in female patients**

**Table S12. The association rules of EPTB and PTB concurrent in <15 years**

**Table S13 The association rules of EPTB and PTB concurrent in 15-25years patients**

**Table S14. The association rules of EPTB and PTB concurrent in 25-35 years patients**

**Table S15. The association rules of EPTB and PTB concurrent in 35-45 years patients**

**Table S16. The association rules of EPTB and PTB concurrent in 45-55 years patients**

**Table S17. The association rules of EPTB and PTB concurrent in 55-65 years patients**

**Table S18. The association rules of EPTB and PTB concurrent in < 65 years patients**

**Table S1.** **The lesion characteristics of EPTB in male patients**

| **lesion characteristics** | **N=1653 (%)** | **Support (%)** | **Confidence (%)** | **Lift** |
| --- | --- | --- | --- | --- |
| **Tuberculous lymphadenitis** | 843(50.99) | 63.23 | 80.05 | 73.23** |
| **lumbar vertebra tuberculosis** | 21(12.70) | 12.71 | 23.09 | 22.22* |
| **Tuberculous pleurisy** | 195(11.79) | 19.34 | 26.10 | 19.13* |
| **Renal tuberculosis** | 10(4.96) | 9.24 | 24.16 | 16.45* |
| **Tuberculous of mediastinal lymph nodes** | 80(4.83) | 32.24 | 32.16 | 32.45* |
| **Tuberculous peritonitis** | 162(9.80) | 43.34 | 43.34 | 43.65* |
| **Intestinal tuberculosis** | 4(2.41) | 5.12 | 12.10 | 9.94* |
| **Chest wall tuberculosis** | 10(4.96) | 9.24 | 24.16 | 19.13* |
| **Bronchial tuberculosis** | 236(14.27) | 27.30 | 27.65 | 27.23* |
| **Musculoskeletal tuberculosis** | 16(9.67) | 10.24 | 10.16 | 10.45* |
| **Tuberculous empyema** | 76(45.97) | 29.12 | 29.34 | 29.65* |

Instances was the cases of the Antecedent. **: strong association rule (lift≥3). *: weak association rule (1≤lift<3).

**Table S2.** **The lesion characteristics of EPTB in female patients**

| **lesion characteristics** | **N=1060(%)** | **Support (%)** | **Confidence (%)** | **Lift** |
| --- | --- | --- | --- | --- |
| **Tuberculous lymphadenitis** | 356(33.58) | 89.10 | 85.94 | 85.87** |
| **lumbar vertebra tuberculosis** | 4(3.77) | 2.05 | 2.25 | 2.44* |
| **Renal tuberculosis** | 6(5.66) | 3.23 | 3.10 | 3.65* |
| **Tuberculous of mediastinal lymph nodes** | 190(17.92) | 31.43 | 31.21 | 31.41** |
| **Tuberculous pleurisy** | 123(11.60) | 22.18 | 21.19 | 21.27* |
| **Bronchial tuberculosis** | 145(13.67) | 25.36 | 25.43 | 25.43* |
| **Tuberculous peritonitis** | 98(9.24) | 19.45 | 19.54 | 19.14* |
| **Tuberculous empyema** | 100(9.43) | 21.68 | 21.32 | 21.19* |
| **Tuberculous meningitis** | 14(13.20) | 7.98 | 7.67 | 7.46* |
| **Chest wall tuberculosis** | 6(5.66) | 3.23 | 3.10 | 2.44* |
| **Intestinal tuberculosis** | 6(5.66) | 3.23 | 3.10 | 2.44* |
| **Musculoskeletal tuberculosis** | 12(11.32) | 8.05 | 8.25 | 8.44* |

Note: Instances were the cases of the Antecedent. **: strong association rule (lift≥3). *: weak association rule (1≤lift<3).

**Table S3. The association rules of concurrent EPTB in <15 years patients with EPTB lesions**

| **lesion characteristics** | **N=**145 **(%)** | **Support (%)** | **Confidence (%)** | **Lift** |
| --- | --- | --- | --- | --- |
| **Tuberculous meningitis** | 14(9.65) | 44.54 | 45.17 | 45.09* |
| **Tuberculous of lymphadenitis** | 57(39.31) | 76.11 | 76.11 | 74.11* |
| **Tuberculous peritonitis** | 17(11.72) | 18.23 | 18.18 | 18.22* |
| **Bronchial tuberculosis** | 13 (8.96) | 17.23 | 17.18 | 17.22* |
| **Tuberculous pleurisy** | 15(10.34) | 15.34 | 14.11 | 14.11* |
| **Tuberculous empyema** | 15(10.34) | 15.56 | 14.54 | 14.43* |
| **Lumbar vertebra tuberculosis** | 14 (9.65) | 17.22 | 17.56 | 17.21* |

Note: Instances were the cases of the Antecedent. **: strong association rule (lift≥3). *: weak association rule (1≤lift<3).

**Table S4. The association rules of EPTB in 15-25 years patients with EPTB lesions**

| **lesion characteristics** | **N=520 (%)** | **Support (%)** | **Confidence (%)** | **Lift** |
| --- | --- | --- | --- | --- |
| **Tuberculous pleurisy** | 58 (11.15) | 53.12 | 63.67 | 63.05* |
| **Tuberculous of lymphadenitis** | 230(44.23) | 75.12 | 70.32 | 70.11** |
| **Tuberculous peritonitis** | 50(9.61) | 53.12 | 63.67 | 63.05* |
| **Tuberculous empyema** | 32(6.15) | 22.56 | 21.34 | 21.35* |
| **Bronchial tuberculosis** | 33(6.34) | 32.12 | 32.32 | 32.11* |
| **Renal tuberculosis** | 26(5.31) | 19.34 | 15.67 | 14.01* |
| **intestinal tuberculosis** | 10(4.88) | 32.09 | 30.00 | 30.00* |
| **lumbar vertebra tuberculosis** | 25(19.23) | 33.56 | 33.34 | 33.35* |
| **Chest wall tuberculosis** | 16(3.07) | 45.34 | 45.72 | 25.01* |
| **Tuberculosis of mediastinal lymph nodes** | 12(2.23) | 49.34 | 45.97 | 24.01* |
| **Musculoskeletal tuberculosis** | 28(5.38) | 10.12 | 39.32 | 29.11* |

Note: Instances were the cases of the antecedent. **: strong association rule (lift≥3). *: weak association rule (1≤lift<3).

**Table S5. The association rules of EPTB in 25-35 years patients with EPTB lesions**

| **lesion characteristics** | **N=300 (%)** | **Support (%)** | **Confidence (%)** | **Lift** |
| --- | --- | --- | --- | --- |
| **Tuberculous pleurisy** | 98(32.66) | 87.45 | 95.36 | 85.53** |
| **Bronchial tuberculosis** | 158(52.66) | 71.89 | 73.67 | 74.67** |
| **Renal tuberculosis** | 23(7.66) | 60.04 | 61.05 | 64.27* |
| **Tuberculous of lymphadenitis** | 21(6.41) | 72.89 | 73.40 | 74.67* |

Note: Instances were the cases of the Antecedent. **: strong association rule (lift≥3). *: weak association rule (1≤lift<3).

**Table S6. The association rules of EPTB in 35-45 years patients with EPTB lesions**

| **lesion characteristics** | **N=675(%)** | **Support (%)** | **Confidence (%)** | **Lift** |
| --- | --- | --- | --- | --- |
| **tuberculous pleurisy** | 100(14.81) | 73.12 | 72.72 | 53.56** |
| **renal tuberculosis** | 98(14.51) | 73.12 | 72.72 | 53.56* |
| **Bronchial tuberculosis** | 87(12.88) | 85.16 | 85.72 | 85.12* |
| **Tuberculosis of lymphadenitis** | 247(32.29) | 76.15 | 76.76 | 76.54* |
| **Tuberculous empyema** | 76(11.25) | 53.12 | 52.72 | 53.56* |
| **tuberculous peritonitis** | 67(9.92) | 33.12 | 32.72 | 33.56* |

Note: Instances were the cases of the Antecedent. **: strong association rule (lift≥3). *: weak association rule (1≤lift<3).

**Table S7. The association rules of EPTB in 45-55 years patients with EPTB lesions**

| **lesion characteristics** | **N=453(%)** | **Support (%)** | **Confidence (%)** | **Lift** |
| --- | --- | --- | --- | --- |
| **Renal tuberculosis** | 54(11.92) | 68.21 | 68.81 | 68.77* |
| **Tuberculous of mediastinal lymph nodes** | 160(35.32) | 52.83 | 50.01 | 51.87** |
| **Tuberculous pleurisy** | 23(5.07) | 30.15 | 33.64 | 33.22* |
| **Bronchial tuberculosis** | 56(13.02) | 42.21 | 43.67 | 43.07* |
| **Tuberculous lymphadenitis** | 90(19.86) | 65.21 | 65.45 | 65.23* |
| **Tuberculous peritonitis** | 70(15.45) | 50.65 | 50.67 | 53.77* |

Note: Instances were the cases of the Antecedent. **: strong association rule (lift≥3). *: weak association rule (1≤lift<3).

**Table S8. The association rules of EPTB in 55-65 years patients with EPTB lesion**s

| **lesion characteristics** | **N=370(%)** | **Support (%)** | **Confidence (%)** | **Lift** |
| --- | --- | --- | --- | --- |
| **tuberculosis of mediastinal lymph nodes** | 98(26.48) | 60.02 | 60.00 | 66.36* |
| **Tuberculous empyema** | 41(11.08) | 62.65 | 63.20 | 62.36* |
| **tuberculous peritonitis** | 23(6.21) | 42.44 | 42.15 | 44.45* |
| **tuberculous lymphadenitis** | 208(56.21) | 72.95 | 72.85 | 74.53** |

Note: Instances were the cases of the Antecedent. **: strong association rule (lift≥3). *: weak association rule (1≤lift<3).

**Table S9. The association rules of EPTB in < 65 years patients with EPTB lesions**

| **lesion characteristics** | **N=250(%)** | **Support (%)** | **Confidence (%)** | **Lift** |
| --- | --- | --- | --- | --- |
| **Bronchial tuberculosis** | 34(13.81) | 14.15 | 14.39 | 15.34* |
| **Tuberculous empyema** | 12(4.40) | 11.15 | 11.14 | 12.34* |
| **Tuberculous peritonitis** | 33(11.11) | 22.12 | 3.39 | 23.12* |
| **Tuberculous of** **lymphadenitis** | 135(44.44) | 74.43 | 70.44 | 70.23** |
| **Tuberculous pleurisy** | 24(7.81) | 7.15 | 7.39 | 7.34** |
| **Chest wall tuberculosis** | 12(4.40) | 11.15 | 11.14 | 12.34* |

Instances were the cases of the Antecedent. **: strong association rule (lift≥3). *: weak association rule (1≤lift<3).

**Table S10. The lesion characteristics of EPTB and PTB concurrent in male patients**

| **lesion characteristics** | **N=4039(%)** | **Support (%)** | **Confidence (%)** | **Lift** |
| --- | --- | --- | --- | --- |
| **Tuberculous lymphadenitis** | 1674 (41.44) | 90.22 | 90.43 | 90.17** |
| **Bronchial tuberculosis** | 222(5.49) | 59.33 | 59.23 | 59.22* |
| **Tuberculous meningitis** | 161(3.98) | 46.26 | 46.35 | 46.13* |
| **Tuberculous pleurisy** | 115(2.84) | 38.21 | 38.35 | 38.64* |
| **Tuberculous peritonitis** | 192(3.38) | 56.61 | 56.35 | 56.54* |
| **Tuberculous empyema** | 102(4.75) | 37.23 | 37.45 | 37.53* |
| **Lumbar vertebra tuberculosis** | 29(0.71 | 13.23 | 15.35 | 13.94* |
| **Tuberculous pericarditis** | 51(1.12) | 19.23 | 15.35 | 13.94* |
| **Tuberculous polyserositis** | 36(2.03) | 15.34 | 15.45 | 15.67* |
| **Intestinal tuberculosis** | 66(0.89) | 18.54 | 18.53 | 18.23* |
| **Chest wall tuberculosis** | 152(3.76) | 44.75 | 44.63 | 44.43* |
| **Tuberculosis of mediastinal lymph nodes** | 151 (3.73) | 42.10 | 42.44 | 42.43* |
| **Renal tuberculosis** | 885(21.91) | 89.76 | 89.38 | 89.43** |
| **Musculoskeletal tuberculosis** | 148(3.66) | 39.02 | 39.11 | 39.13* |

Instances were the cases of the Antecedent. **: strong association rule (lift≥3). *: weak association rule (1≤lift<3).

**Table S11. The lesion characteristics of EPTB and PTB concurrent in female**

| **lesion characteristics** | **N=4093(%)** | **Support (%)** | **Confidence (%)** | **Lift** |
| --- | --- | --- | --- | --- |
| **Tuberculous lymphadenitis** | 2908(71.99) | 88.65 | 88.91 | 88.23** |
| **Bronchial tuberculosis** | 324(7.91) | 34.12 | 34.51 | 43.54* |
| **Tuberculous meningitis** | 210(5.13) | 29.54 | 29.54 | 29.55* |
| **Tuberculous pleurisy** | 185(4.51) | 25.32 | 25.31 | 25.35* |
| **Tuberculous peritonitis** | 145(3.54) | 20.22 | 20.43 | 20.34* |
| **Tuberculous empyema** | 210(5.13) | 29.54 | 29.54 | 29.55* |
| **Lumbar vertebra tuberculosis** | 200(4.76) | 28.43 | 28.34 | 28.65* |
| **Tuberculous pericarditis** | 33(0.80) | 9.86 | 9.56 | 9.14* |
| **Tuberculous polyserositis** | 100(2.38) | 15.76 | 15.91 | 15.23* |
| **Intestinal tuberculosis** | 100(2.38) | 15.76 | 15.91 | 15.23* |
| **Chest wall tuberculosis** | 210(5.13) | 29.54 | 29.54 | 29.55* |
| **Tuberculosis of mediastinal lymph nodes** | 321(7.84) | 31.23 | 31.22 | 31.23* |
| **Renal tuberculosis** | 965(23.89) | 76.39 | 76.16 | 76.14** |
| **Musculoskeletal tuberculosis** | 119(2.94) | 17.21 | 17.32 | 17.43* |

Instances were the cases of the Antecedent. **: strong association rule (lift≥3). *: weak association rule (1≤lift<3).

**Table S12. The association rules of EPTB and PTB concurrent in <15 years**

| **lesion characteristics** | **N=1467 (%)** | **Support (%)** | **Confidence (%)** | **Lift** |
| --- | --- | --- | --- | --- |
| **Tuberculous meningitis** | 225(15.33) | 70.23 | 70.21 | 70.12** |
| **Tuberculous pleurisy** | 43(2.93) | 34.34 | 34.22 | 34.10* |
| **Tuberculous of mediastinal lymph nodes** | 432(29.44) | 87.12 | 88.99 | 88.12** |
| **Tuberculous peritonitis** | 98(6.68) | 0.21 | 1.17 | 1.13* |
| **Tuberculous empyema** | 55(3.74) | 44.87 | 44.87 | 42.25* |
| **Bronchial tuberculosis** | 322(21.94) | 76.05 | 76.69 | 76.88** |
| **Tuberculous lymphadenitis** | 245(16.70) | 72.32 | 72.19 | 72.25* |
| **Intestinal tuberculosis** | 27(1.84) | 23.76 | 22.99 | 23.12* |
| **lumbar vertebra tuberculosis** | 20(1.35) | 20.18 | 20.15 | 20.55* |

Note: Instances were the cases of the antecedent. **: strong association rule (lift≥3). *: weak association rule (1≤lift<3).

**Table S13 The association rules of EPTB and PTB concurrent in 15-25 years patients**

| **lesion characteristics** | **N=1334(%)** | **Support (%)** | **Confidence (%)** | **Lift** |
| --- | --- | --- | --- | --- |
| **Tuberculous meningitis** | 126(17.64) | 34.43 | 34.87 | 34.24* |
| **Tuberculous pleurisy** | 115(14.70) | 30.00 | 30.22 | 30.23* |
| **Tuberculous of mediastinal lymph nodes** | 117(20.58) | 31.12 | 31.09 | 31.10* |
| **Tuberculous** **lymphadenitis** | 600(17.64) | 77.23 | 76.85 | 75.45** |
| **Tuberculous peritonitis** | 100(2.94) | 23.12 | 22.13 | 21.15* |
| **Tuberculous empyema** | 120(5.88) | 32.15 | 32.14 | 32.06* |
| **Bronchial tuberculosis** | 80(5.88) | 29.16 | 29.11 | 29.28* |
| **Tuberculous pericarditis** | 34(5.88) | 12.65 | 12.54 | 12.58* |
| **Intestinal tuberculosis** | 23(5.88) | 10.54 | 10.54 | 12.32* |
| **lumbar vertebra tuberculosis** | 19(2.94) | 8.14 | 8.45 | 8.14* |

Note: Instances was the cases of the antecedent. **: strong association rule (lift≥3). *: weak association rule (1≤lift<3).

**Table S14. The association rules of EPTB and PTB concurrent in 25-35 years patients**

| **lesion characteristics** | **N=** **1481 (%)** | **Support (%)** | **Confidence (%)** | **Lift** |
| --- | --- | --- | --- | --- |
| **Tuberculous meningitis** | 20(1.35) | 20.12 | 20.11 | 20.24* |
| **Tuberculous pleurisy** | 32(2.16) | 15.45 | 15.88 | 10.12* |
| **Tuberculous of mediastinal lymph nodes** | 32(2.16) | 15.45 | 15.88 | 10.12* |
| **Tuberculous empyema** | 23(1.55) | 9.45 | 9.73 | 9.21* |
| **tuberculous peritonitis** | 28(1.89) | 10.34 | 10.03 | 10.97* |
| **Lumbar vertebra tuberculosis** | 24(1.62) | 10.34 | 10.03 | 10.97* |
| **bronchial tuberculosis** | 27(1.82) | 13.64 | 12.88 | 12.12* |
| **Tuberculous lymphadenitis** | 991(66.91) | 86.87 | 88.86 | 88.78** |
| **Renal tuberculosis** | 120(8.10) | 29.54 | 29.55 | 29.55* |
| **Musculoskeletal tuberculosis** | 20(1.35) | 30.42 | 30.41 | 30.12* |
| **Tuberculous polyserositis** | 20(1.35) | 12.56 | 12.03 | 12.97* |
| **Chest wall tuberculosis** | 23(1.55) | 34.55 | 31.45 | 30.78* |
| **Tuberculous pericarditis** | 50(3.37) | 23.14 | 23.12 | 23.17* |
| **Intestinal tuberculosis** | 80(5.40) | 25.16 | 25.11 | 25.12* |

Note: Instances were the cases of the antecedent. **: strong association rule (lift≥3). *: weak association rule (1≤lift<3).

**Table S15. The association rules of EPTB and PTB concurrent in 35-45 years patients**

| **lesion characteristics** | **N=1786(%)** | **Support (%)** | **Confidence (%)** | **Lift** |
| --- | --- | --- | --- | --- |
| **Musculoskeletal tuberculosis** | 125(6.99) | 34.32 | 34.46 | 32.66* |
| **Tuberculous pleurisy** | 111(6.21) | 21.34 | 20.59 | 20.43* |
| **Tuberculous of mediastinal lymph nodes** | 122(6.83) | 32.45 | 31.46 | 31.66* |
| **Tuberculous lymphadenitis** | 600(33.72) | 86.56 | 86.44 | 86.15** |
| **Tuberculous peritonitis** | 111(6.21) | 21.34 | 20.59 | 20.43* |
| **Tuberculous empyema** | 114(6.38) | 32.11 | 32.11 | 32.11* |
| **bronchial tuberculosis** | 117(6.55) | 34.19 | 34.56 | 34.43* |
| **Chest wall tuberculosis** | 116(7.80) | 33.13 | 33.16 | 33.78* |
| **intestinal tuberculosis** | 116(7.80) | 33.13 | 33.16 | 33.78* |
| **lumbar vertebra tuberculosis** | 15(9.83) | 10.00 | 10.59 | 9.35* |
| **Renal tuberculosis** | 121(6.71) | 38.46 | 37.47 | 37.66* |
| **Tuberculous polyserositis** | 116(7.80) | 33.13 | 33.16 | 33.78* |

Note: Instances were the cases of the antecedent. **: strong association rule (lift≥3). *: weak association rule (1≤lift<3).

**Table S16. The association rules of EPTB and PTB concurrent in 45-55 years patients**

| **lesion characteristics** | **N=1656(%)** | **Support (%)** | **Confidence (%)** | **Lift** |
| --- | --- | --- | --- | --- |
| **Intestinal tuberculosis** | 322(19.44) | 49.31 | 49.12 | 49.10* |
| **Chest wall tuberculosis** | 223(14.97) | 43.14 | 44.39 | 43.96* |
| **Tuberculous lymphadenitis** | 990(59.78) | 89.23 | 89.82 | 89.65** |
| **Lumbar vertebra tuberculosis** | 121(7.30) | 29.37 | 29.24 | 29.64* |

Note: Instances were the cases of the antecedent. **: strong association rule (lift≥3). *: weak association rule (1≤lift<3).

**Table S17. The association rules of EPTB and PTB concurrent in 55-65 years patients**

| **lesion characteristics** | **N=1726(%)** | **Support (%)** | **Confidence (%)** | **Lift** |
| --- | --- | --- | --- | --- |
| **Tuberculous lymphadenitis** | 847(49.07) | 89.45 | 89.13 | 89.23** |
| **Renal tuberculosis** | 879(50.92) | 89.34 | 89.54 | 89.12** |

Note: Instances were the cases of the antecedent. **: strong association rule (lift≥3). *: weak association rule (1≤lift<3).

**Table S18. The association rules of EPTB and PTB concurrent in** **< 65 years patients**

| **lesion characteristics** | **N=673 (%)** | **Support (%)** | **Confidence (%)** | **Lift** |
| --- | --- | --- | --- | --- |
| **Renal tuberculosis** | 338(50.22) | 58.23 | 58.22 | 58.33** |
| **Lumbar vertebra tuberculosis** | 100(14.85) | 39.56 | 39.45 | 39.31* |
| **Tuberculous lymphadenitis** | 124(18.42) | 42.12 | 42.34 | 42.23* |
| **Intestinal tuberculosis** | 111(16.49) | 41.12 | 41.34 | 41.23* |

Note: Instances were the cases of the antecedent. **: strong association rule (lift≥3). *: weak association rule (1≤lift<3).
